# Assessing chlorhexidine resistance in MRSA isolates from hospitals in Cleveland, OH and Detroit, MI

**DOI:** 10.1017/ash.2024.270

**Published:** 2024-09-16

**Authors:** Collin Telchik, Chetan Jinadatha, Jennifer Cadnum, Curtis Donskey, Piyali Chatterjee, Sorabh Dhar, Keith Kaye, Taylor Yakubik, Munok Hwang, Andrea Grimbergen

**Affiliations:** Department of Internal Medicine, Baylor Scott and White Medical Center, Temple, Texas; Central Texas Veterans Health Care System; Cleveland VA Medical Center; Harper Unive Hosp; Rutgers Robert Wood Johnson Medical School; Baylor Scott and White; Central Texas VA Research Foundation

## Abstract

**Background:** Methicillin-resistant Staphylococcus aureus (MRSA) is one of the most common causes of procedure-related, skin, and soft-tissue infections. Hospitalized patients who are colonized with MRSA are at a higher risk of developing invasive infections after discharge. Chlorhexidine, an antiseptic/disinfectant, has been used to reduce carriage and prevent infections in these patients. Studies have shown chlorhexidine resistance among MRSA strains. Chlorhexidine resistance is associated with qac genes, which encode multidrug efflux pumps that increase bacterial tolerance to disinfectant agents. The global distribution and prevalence of qacA and qacB genes are highly variable. One study reported that qacA and qacB genes could be found in 0.9% - 83.3% of clinical MRSA isolates worldwide. The goal of this study was to determine the prevalence of chlorhexidine resistance and identify the resistance-associated genes from our MRSA samples using whole genome sequencing (WGS). **Methods:** 474 MRSA samples were obtained from hospitals in Detroit, MI (287) and Cleveland, OH (187). Whole genome sequencing was performed using the NextSeq (Illumina Inc., CA) platform. The sequencing data was analyzed using ResFinder 4.1, a publicly available database that can be used to identify acquired genes and chromosomal mutations mediating antimicrobial resistance. The output was organized into a data sheet to visualize the presence of the genes of interest. **Results:** The qacA gene was present in only one MRSA sample from the Cleveland area hospital. In the samples from Detroit, 14 out of 287 showed disinfectant resistance genes. The qacA, qacB, and qacD were present in 1, 6, and 7 samples, respectively. The prevalence of any qac gene in the Cleveland area samples was 0.5%. Meanwhile, the prevalence of any qac gene in Detroit area samples was 4.9%. Among the 7 samples that have qacD gene, 6 samples have more than one copy of qacD. **Conclusions:** The prevalence of the “qac” gene varied widely based on the origin of the samples. Detroit area samples had more qac genes prevalence than Cleveland area samples. Chlorhexidine is a widely used antiseptic/disinfectant, and it plays a vital role in reducing carriage and preventing infection among hospitalized patients colonized with MRSA. Monitoring and addressing MRSA-reduced susceptibility to chlorhexidine is imperative for maintaining the effectiveness of infection control practices such as decolonization.

